# Immediate effect of CPAP titration on perceived health related quality of life: a prospective observational study

**DOI:** 10.1186/s12890-016-0336-8

**Published:** 2016-12-01

**Authors:** Serena Iacono Isidoro, Adriana Salvaggio, Anna Lo Bue, Salvatore Romano, Oreste Marrone, Giuseppe Insalaco

**Affiliations:** National Research Council of Italy, Institute of Biomedicine and Molecular Immunology “A. Monroy”, Via Ugo La Malfa, 153, Palermo, 90146 Italy

**Keywords:** Obstructive sleep apnea, Quality of Life, CPAP titration, Gender

## Abstract

**Background:**

Perceived Health Related Quality of Life (HRQoL) is impaired in obstructive sleep apnea (OSA). This study examines changes in HRQoL aspects occurring immediately after CPAP titration. Furthermore, we analyzed variations in each gender and in patients undergoing home or laboratory-based CPAP titration pathways.

**Methods:**

Twohundredfive outpatients (151 M) (56.7 ± 10.3 years) were evaluated, before first visit and nocturnal diagnostic examination (T0), and the morning after CPAP titration (T1). Two self-reported HRQoL questionnaires were administered: Psychological General Well-Being Index (PGWBI), composed by six subscales, and 12-Item Short-Form Health Survey (SF-12), including Physical (PCS) and Mental Component Summaries (MCS). CPAP titration was performed using auto-adjusting CPAP units at patients’ home or in the sleep laboratory.

**Results:**

PGWBI scores at T1 improved compared to T0 (*p* < 0.0001). A similar improvement was observed in SF-12 MCS (*p* = 0.0011), but not in SF-12 PCS. Changes were independent from anthropometric parameters, OSA severity and excessive daytime sleepiness. Gender comparisons showed better HRQoL in males at both times. At T0, patients who received home or laboratory CPAP titration pathways did not show any differences in PGWBI and SF-12 scores. At T1, PGWBI and SF-12 MCS improved in both home and laboratory groups.

**Conclusions:**

This study gives evidence that first time CPAP application for titration can lead to a general increase in perceived well-being. Gender comparisons showed better perceived HRQoL with more subscales improvements in males after CPAP titration. The improvement was similar with both home and laboratory CPAP titration pathways.

## Background

Obstructive sleep apnea (OSA) is a common disorder characterized by repetitive episodes of complete (apnea) or partial (hypopnea) upper airway obstruction during sleep, associated with ongoing increasing respiratory efforts, intermittent hypoxia, systemic and pulmonary arterial blood pressure fluctuations and sleep disruption. Approximately 9% of females and 24% of men in the general population have OSA [1] and most remain undiagnosed.

OSA patients may exhibit several typical symptoms including habitual snoring (often disruptive for bed partners), feeling of unrefreshed awaking, headache, neurocognitive deficit, fatigue, excessive daytime sleepiness (EDS). These symptoms have often a significant impact on health-related quality of life (HRQoL) including its social and psychological domains [2–6].

There is a number of effective treatment options for OSA (including lifestyle advice, mandibular splint devices, surgery, etc.); however, the current most widely prescribed therapy for patients with moderate-severe OSA or mild OSA with comorbidities is nocturnal continuous positive airway pressure (CPAP). CPAP is usually set at a pressure sufficient to prevent upper airway collapse in all body positions and sleep stages. CPAP titration is determined by an attended overnight CPAP titration study with full polysomnographic (PSG) monitoring in a sleep laboratory, or by automatic positive airway pressure (APAP) devices [7–9].

OSA treatment with CPAP has shown to improve HRQoL [6]. However the initial impact of CPAP on many aspects related to HRQoL like mood, psychological well-being, self-control, energy, mental and physical health and fatigue have not been investigated.

Aim of this study was to evaluate the immediate effect of CPAP administration on self-perceived HRQoL in OSA patients referred to a laboratory for sleep related breathing disorders. Furthermore, we analyzed HRQoL-related variations in each gender and in patients undergoing home or laboratory CPAP titration pathways. We have already evaluated the HRQoL of patients at the first time visit [10], and after diagnosis disclosure [11]. What makes this trial unique and distinguishes it from other such studies is that, as far as we know, for the first time it examines the immediate effect of CPAP on perceived well-being.

## Methods

We performed a study involving patients afferent to our Sleep Laboratory for respiratory disorders during sleep. Out of 1067 consecutive outpatients, 205 completed the study (54 F, 151 M), age 25–79 years (56.7 ± 10.3).

This was a prospective observational study. All CPAP-naïve patients with a new diagnosis of OSA were eligible to be included in this study.

**Fig. 1:**
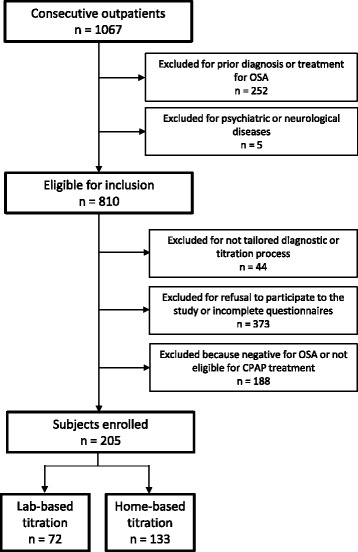
Cohort diagram of participants to the study enrolled for the home and in-laboratory management pathways

The exclusion criteria were: refusal to participate, refusal of CPAP therapy, central sleep apnoea syndromes, obesity hypoventilation syndrome, restrictive pulmonary and restrictive chest wall diseases, severe congestive heart failure, a history of life-threatening arrhythmias, severe cardiomyopathy, long-term oxygen therapy, drug or alcohol abuse, severe cognitive impairment, concurrent oncological diseases, and a history of narcolepsy or restless legs syndrome. Patients affected by psychiatric or neurological disease were also excluded, as well as subjects taking neurological medication. Patients with a prior diagnosis or treatment for OSA were excluded (n. 252), as were subjects who did not complete full diagnostic and CPAP titration process or questionnaires (n. 605) (Fig. 1).

Subjects underwent a detailed clinical evaluation that included sleep-related disorders. Institutional Ethical Committee approved the protocol (Palermo I AOUP “P. Giaccone”, report no 8/2014) and all subjects gave their written informed consent for personal data processing.

Nocturnal monitoring was performed with a portable computerized system (Somté or Somnea Compumedics Inc.; Abbotsford, VIC, Australia). The recorded signals were airflow, snoring, thoracic and abdominal movements, limb movements, body position, arterial oxygen saturation, pulse rate, and pulse waveform. Duration of recordings was at least 6 h. Apneas and hypopneas were visually scored and OSA severity defined according to American Academy of Sleep Medicine standard criteria [12]. Percent study-time with O_2_ saturation <90% (TSat90) was evaluated.

### Questionnaires

The Psychological General Well-Being Index (PGWBI) was used to measure perceived psychological well-being. The responses to 22 questions are arranged in six subscales: Anxiety, Depression, Well-being, Self-control, Health and Vitality. Item responses are rated on a six-point Likert scale ranging from 0 to 5. Higher scores indicate better well-being. The subscales sum provides a global index score for subjective well-being (range 0–110). Considering “distress” as the reverse of well-being, a global score <60 suggests a “Severe Distress”; from 60 to 72 a “Moderate Distress”; and >72 “No Distress” category [13, 14].

The 12-Item Short-Form Health Survey (SF-12) provides a Physical Component Summary (PCS) and a Mental Component Summary (MCS). The PCS and MCS are standardised to a mean of about 50 (range 0–100), with a score above 50 representing better than average function and below 50 poorer than average function [15, 16].

The Italian version of Epworth Sleepiness Scale (ESS) [17, 18] was administered to assess daytime sleepiness. A score > 10 suggests EDS.

### Study procedure

Patients’ HRQoL was assessed at the first visit (T0), and the morning after CPAP titration (T1). A portion of the sample was included in previous studies [10, 11].

The study affected neither the patients’ diagnostic procedure and routine care nor the timing of CPAP titration.

After diagnosis, patients received counseling by a sleep physician regarding the diagnostic results, basic information on OSA, its effects on comorbid conditions and daytime performance, the importance of treatment adherence and the consequences of ineffective treatment.

Then, the staff identified the suitable mask and held a session of education and training to CPAP therapy and mask fitting. The duration of this phase was about 30 min. Subjects were allowed to choose to receive CPAP titration at home or in the laboratory. Titration was performed using auto-adjusting CPAP units (S9TM, ResMed Ltd, Bella Vista, Australia) at patients’ home for 3 to 5 nights or in the sleep lab . Throughout the night and the next morning, the staff on duty in the lab dealt with any discomfort related to the CPAP treatment. The pressure selected for CPAP treatment was the pressure at which the participant spent 90% of the time with apnea-hypopnea index (AHI) lower than 10 events/hour [8]. Successful unattended titration of APAP require a minimum of 1 night with at least 6 h of total recording, and at least 5 h with a mean mask leak <0.4 l/s [9]. We considered the home titration acceptable if it was carried out ≥2 nights, its average duration was ≥ 4 h/night and leaks were <24 l/min.

In order to explore whether, and to what extent, CPAP titration affects patients’ HRQoL, an assistant psychologist administered self-reported questionnaires in the same order: ESS only at T0, and PGWBI, followed by SF-12 at T0 and T1.

### Statistical analysis

Data are reported as mean ± SD. The non-parametric Wilcoxon test was used to assess differences between genders, smoking, alcohol use, comorbidities, OSA severity and between laboratory and home groups in terms of characteristics and HRQoL scores. Questionnaires mean differences between T0 and T1 were evaluated by paired *t*-test. The Cohen’s *d* effect size (ES), for the two paired samples (T0, T1), was estimated dividing the *t* value of the paired *t*-test by the square root of the number of pairs. Effects sizes show the magnitude of differences observed [19].

Pearson’s chi-square was applied to assess differences in “Distress category” between T0 and T1. Relationships between age, BMI, AHI, Tsat90, ESS and HRQoL scores changes (scores at T1 minus scores at T0) were identified through a linear regression analysis. A *p* < 0.05 was considered significant. Statistical analysis was performed by commercial software (JMP 8.0 SAS Institute Inc.).

## Results

Sample characteristics and nocturnal sleep study results for all subjects and by gender are shown in Table 1. Male and female patients differed in age and body mass index (BMI). In our sample, 47 subjects (22,9%) were smokers, and 108 subjects (52,7%) had hypertension.

**Table 1:** Patients characteristics and nocturnal polygraphic results

	Total	Female	Male
Subjects, No.	205	54	151
Age, yrs	56.7 ± 10.3 (25–79)	59.8 ± 7.8 (42–76)	55.6 ± 10.9* (25–79)
BMI, kg/m^2^	33.0 ± 6.4 (22.8–51.6)	35.8 ± 7.7 (22.8–51.6)	32.0 ± 5.6** (23.0–50.0)
AHI, n/h	47.8 ± 24.4 (6–118)	48.1 ± 29.0 (9–118)	47.6 ± 22.7 (6–101)
Mild, No. (%)	18 (8.8)	7 (13.0)	11 (7.3)
Moderate, No. (%)	40 (19.5)	11 (20.4)	29 (19.2)
Severe, No. (%)	147 (71.7)	36 (66.6)	111 (73.5)
TSat_90,_ %	24.4 ± 23.9 (0–93.1)	29.2 ± 27.6 (0.1–93)	22.6 ± 22.3 (0–93.1)
ESS score	11.0 ± 5.0 (1–24)	10.9 ± 5.0 (2–22)	11.0 ± 5.1 (1–24)

Data are given as mean ± SD (range). BMI = body mass index; AHI = Apnea hypopnea index; Mild = AHI ≥5, but < 15per hour; Moderate = AHI ≥15, but < 30per hour; Severe = AHI ≥30 per hour; TSat_90_ = percent study-time at less than 90% oxygen saturation; ESS = Epworth Sleepiness Scale; F = Female; M = Male

*p = 0.0131, **p = 0.0018 F vs M

Table 2 shows PGWBI and SF-12 sample scores at T0 and T1. In the whole sample, at T0 mean questionnaires scores were worse than reference values (*p* < 0.0260) [13, 15]. At T1, PGWBI scores were improved compared to T0, and Anxiety, Depression and Well-Being PGWBI subscales became similar to reference values. SF-12 showed an improvement for MCS, but not for PCS. ES indicated medium levels of improvement for PGWBI total score, and Anxiety, Depression, Health and Vitality subscales. Linear regression analysis between questionnaires changes (scores at T1 minus scores at T0), and age, BMI, AHI, Tsat90 and ESS did not show any significant relationship.

**Table 2:** Health-related quality of life data by PGWBI and SF-12

	T0	T1	ES
PGWBI	69.5 ± 16.8	74.7 ± 17.4*	0.45
Anxiety	15.7 ± 4.8	17.4 ± 4.7*	0.42
Depression	12.0 ± 2.6	12.6 ± 2.3*	0.30
Well-being	11.1 ± 3.9	11.9 ± 3.8**	0.25
Self-control	10.8 ± 3.0	11.1 ± 3.0†	0.14
Health	9.3 ± 2.8	10.1 ± 2.6*	0.37
Vitality	10.6 ± 3.8	11.6 ± 3.7*	0.31
SF-12 PCS	42.7 ± 9.8	43.2 ± 10.2	-
SF-12 MCS	44.8 ± 10.4	46.7 ± 10.6**	0.23

Data are given as mean ± SD. PGWBI = Psychological General Well-Being Index; SF-12 = 12-Item Short-Form Health Survey; PCS = Physical Component Summary; MCS = Mental Component Summary; T0 = first time visit; T1 = after CPAP titration; ES = Effect Size

*p < 0.0001, **p < 0.005, †*p* < 0.05 T0 vs T1

Characteristics of populations in terms of smoking, alcohol use, comorbidities and analysis stratified by OSA severity did not show any significant relationship with HRQoL score changes.

Table 3 shows PGWBI and SF-12 scores for each gender. Mean questionnaires scores were better for males compared to females at both times (*p* < 0.0460), except for Anxiety PGWBI subscale at T0. Females showed a medium ES improvement in PGWBI total score and Anxiety, Health and Vitality subscales, and SF-12 MCS. Males improved all scores except for Self-control PGWBI subscale and SF-12 PCS, with a medium ES on PGWBI Total score and Anxiety, Depression and Health subscales (Table 3).

**Table 3:** Health-related quality of life data by PGWBI and SF-12 for gender

	Female (54)			Male (151)		
	T0	T1	ES	T0	T1	ES
PGWBI	61.7 ± 19.5	67.7 ± 19.1**	0.40	72.3 ± 14.9	77.2 ± 15.8*	0.48
Anxiety	14.4 ± 5.8	16.1 ± 5.0†	0.33	16.1 ± 4.4	17.8 ± 4.6*	0.46
Depression	11.3 ± 2.9	11.6 ± 2.8	-	12.2 ± 2.4	13.0 ± 2.0*	0.38
Well-being	9.3 ± 4.1	10.0 ± 3.9	-	11.8 ± 3.6	12.5 ± 3.6**	0.28
Self-control	9.3 ± 3.5	9.8 ± 3.5	-	11.3 ± 2.6	11.6 ± 2.7	-
Health	8.0 ± 2.9	9.4 ± 2.8**	0.51	9.7 ± 2.6	10.4 ± 2.5**	0.31
Vitality	9.3 ± 4.0	10.7 ± 4.1†	0.35	11.1 ± 3.5	12.0 ± 3.5**	0.29
SF-12 PCS	36.7 ± 9.4	38.0 ± 9.6	-	44.8 ± 9.0	45.0 ± 9.7	-
SF-12 MCS	40.7 ± 11.3	43.9 ± 10.9†	0.36	46.3 ± 10.3	47.7 ± 10.3†	0.18

Data are given as mean ± SD. PGWBI = Psychological General Well-Being Index; SF-12 = 12-Item Short-Form Health Survey; PCS = Physical Component Summary; MCS = Mental Component Summary; T0 = first time visit; T1 = after CPAP titration; ES = Effect Size; F = Female; M = Male

*p < 0.0001, **p < 0.005, †*p* < 0.05 T0 vs T1

**Fig. 2:**
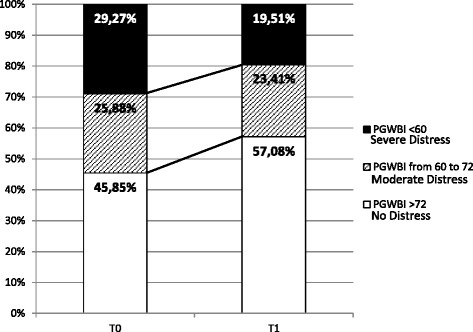
Sample distribution for PGWBI Total before and after titration. T0 = first time visit; T1 = after CPAP titration. Higher scores indicate better well-being. Chi-square = 110.72; *p* < 0.0001. At T0: 60 subjects had PGWBI <60; 51 subjects had PGWBI from 60 to 72; 94 subjects had PGWBI >72. At T1: 40 subjects had PGWBI <60; 48 subjects had PGWBI from 60 to 72; 117 subjects had PGWBI >72

Figure 2 shows sample distribution for PGWBI Total score at both times, as reported in PGWBI Manual [13]. Category distribution was different between T0 and T1 (chi-square = 110.72; *p* < 0.0001), with an increase in subjects with PGWBI Total score >72 after CPAP titration.

Dividing the sample in laboratory and home CPAP titration pathways, the characteristics of both groups were similar. Comparing PGWBI and SF-12 scores at T0 and T1 separately for laboratory and home CPAP titration pathways, we found differences between groups at T0 for Well-being (*p* = 0.0288) and Vitality (*p* = 0.0354) PGWBI subscales, and for SF-12 MCS (*p* = 0.0464). At T1 HRQoL scores of laboratory and home CPAP titration pathways, were not different.

At T1, the home group improved in all scores except one (SF-12 PCS), while laboratory group improved in Anxiety, Depression and Health PGWBI subscales. ES indicated medium changes for home group in PGWBI total score, and Anxiety, Depression, Well-being, Health and Vitality subscales, and in SF-12 MCS.

## Discussion

The results of this study show that HRQoL among subjects afferent to our sleep center for suspicion of OSA was impaired at first visit. CPAP titration was followed by a significant improvement in HRQoL as evaluated by PGWBI and SF-12, irrespective of age, BMI, OSA severity, nocturnal hypoxia and EDS. Sample distribution for PGWBI scores at T1 showed an increase of subjects with the highest well-being. Males showed better scores than females in both questionnaires at T0 and at T1. After CPAP titration, patients receiving home and laboratory CPAP titration pathways achieved similar scores in HRQoL questionnaires. Home group improved in every questionnaires subscale, except SF-12 PCS, while those with laboratory CPAP titration showed a significant improvement only in Anxiety, Depression and Health PGWBI subscales.

OSA is a very prevalent still largely under-diagnosed disease that significantly impairs quality of life [3–5]. Many studies highlighted that untreated patients with OSA have a worse quality of life when compared with the general population [20]. Similarly to the present study, other investigators have observed no correlation between OSA severity and HRQoL, emphasizing that the physiological parameters alone may not be the only determinants of HRQoL [21, 22]. The consequences of sleep-related breathing disorders on HRQoL may be considered as a multifactorial phenomenon, including not only sleep disruption, sleepiness and obesity, but also mood and psychosocial factors, which are all implicated in different aspects of physical and mental health perception [23].

As far as we know, there are no studies that examine perceived HRQoL variation immediately after CPAP titration. Nevertheless, many studies have analyzed the effect of 3–6 months of CPAP therapy on HRQoL, pointing out a significant improvement [24]. In our study, the improvement on perceived HRQoL is noticeable immediately after CPAP titration. Besides, it is possible to hypothesize that, after long-term CPAP use, the change in HRQoL would be even more evident and extended to more dimensions (for instance, those relating to SF-12 PCS), not yet impacted after CPAP titration.

In our sample, males’ perceived HRQoL was better than females’, and both genders showed an improvement after CPAP titration. Male subjects reported a significant improvement in SF-12 MCS and in all PGWBI subscales except for Self-Control, while females improved only in total PGWBI score and Health and Vitality subscales. However, adequately powered studies are necessary to examine gender differences in OSA clinical manifestations and response to CPAP. Gender differences could be explained by female characteristics such as greater bodily attention, as well as social acceptance for females to express distress [25]. Females in a healthy population reported poorer well-being and had a higher symptom complaint rate [13, 15]. Furthermore, other studies suggest that females have more psychological morbidity and a poorer HRQoL than men in association with OSA [26], that may be due to differences in experience of OSA, like insomnia, depression vs. sleepiness, irritability.

Another finding of the present study is that both patients receiving home and laboratory CPAP titration perceived an improvement in HRQoL. Similar results were showed in previous reports that compared patients HRQoL assessed before laboratory or home CPAP titration and at 1 or 3 months follow-up visit, finding no statistical differences between groups [27–29].

Our results show that benefits on HRQoL perception for home pathway are non-inferior to those with laboratory CPAP titration, underlining also some advantage for home CPAP titration and suggesting that comfortable environment may be useful to increase patients’ opportunity to experience CPAP benefits.

Many studies demonstrated that home unattended diagnostic/therapeutic program is a practical strategy, as effective as the laboratory attended evaluation, resulting in similar functional outcomes and adherence to CPAP treatment [7, 8, 28]. In addition, it has proved more comfortable for patients, avoiding the “first-night effect”, generally not present at home, that could induce variability in sleep architecture [30]. Moreover, CPAP therapy needs more cooperation than just taking a pill. Several studies have underlined the importance of the impact of first feeling with CPAP use as significant predictor to future treatment adherence, suggesting that benefits perceived after first night of CPAP titration are relevant in determining CPAP adherence [30–32]. A better information about OSA [33, 34], and a first experience with CPAP in a comfortable environment may increase confidence in health staff and compliance to the CPAP treatment, and improve doctor-patient relationship.

Our study has some limitations. One concerns the difference in the number of days of CPAP titration between home and laboratory groups. However, our main outcome was the immediate effect of CPAP and we chose to compare a 3 to 5-night period of home titration with a 1-night laboratory titration to guarantee the comfort for patients who were living far away from the sleep laboratory and to compare the effects of two CPAP titration pathways, as performed routinely, in order to appreciate differences, if any, between common methods of patients management. Furthermore, subjects were allowed to choose to receive CPAP titration at home or in the laboratory, therefore, there may be an unmeasured self-selection bias whereby subjects who chose to have home titrations may have been more likely to have perceived improvement in HRQoL. Another limitation was related to the different size of males and females subgroups. Further studies are needed to assess gender differences. Furthermore, we used the SF-12, whereas using SF-36 could have potentially provided better discrimination. However, PGWBI and SF-12 were administered together to guarantee a further extension of HRQoL measures, avoiding an excessive and confusing number of items during administration.

Despite these limitations, the study provides evidence that CPAP titration has an impact on perceived well-being, and that HRQoL improves as soon as CPAP is initiated. We can also address an important clinical question about home CPAP titration benefits that are not inferior to laboratory titration pathway.

## Conclusions

In summary, this study shows that CPAP titration can lead to a general increase in perceived well-being, regardless of age, gender, BMI, AHI, TSat90 and EDS. Gender comparisons showed better perceived HRQoL with more subscales improvements in males after CPAP titration. After CPAP titration, regardless of home or laboratory pathway, there was an improvement in perceived HRQoL. The first night may improve the feeling to CPAP therapy, underlining that development of innovative medical interventions in comfortable home environment is effective to improve patients’ opportunity to adapt and experience CPAP benefits. New studies are required to evaluate the influence of perceived HRQoL after CPAP titration on the subsequent adherence to CPAP treatment.

## Abbreviations

AHI: Apnea hypopnea index
BMI: Body mass index
CPAP: Continuous positive airway pressure
EDS: Excessive daytime sleepiness
ES: Effect size
ESS: Epworth sleepiness scale
HRQoL: Health related quality of life
MCS: SF-12 Mental Component Summary
OSA: Obstructive Sleep Apnea
PCS: SF- 12 Physical Component Summary
PGWBI: Psychological General Well-Being Index
SF-12: 12-Item Short-Form Health Survey
T0: Before first visit and nocturnal diagnostic examination
T1: The morning after CPAP titration
TSat90: Percent study-time at less than 90% oxygen saturation
